# Passion fruit seed extract protects beta‐amyloid‐induced neuronal cell death in a differentiated human neuroblastoma SH‐SY5Y cell model

**DOI:** 10.1002/fsn3.2757

**Published:** 2022-02-02

**Authors:** Akira Sato, Nozomi Tagai, Yoko Ogino, Haruka Uozumi, Shinpei Kawakami, Takayuki Yamamoto, Sei‐ichi Tanuma, Hiroko Maruki‐Uchida, Sadao Mori, Minoru Morita

**Affiliations:** ^1^ Department of Biochemistry and Molecular Biology Faculty of Pharmaceutical Sciences Tokyo University of Science Noda, Chiba Japan; ^2^ Department of Biochemistry Faculty of Pharmaceutical Sciences Tokyo University of Science Noda, Chiba Japan; ^3^ Research and Development Institute Health Science Research Center, Morinaga & Co., Ltd. Yokohama Japan; ^4^ Department of Genomic Medicinal Science Research Institute for Science and Technology Organization for Research Advancement Tokyo University of Science Noda, Chiba Japan; ^5^ Present address: Department of Gene Regulation Faculty of Pharmaceutical Sciences Tokyo University of Science Noda, Chiba Japan

**Keywords:** Alzheimer's disease, functional food, neurite fragmentation, neuronal cell death, passion fruit seed extract, piceatannol

## Abstract

Alzheimer's disease (AD) is a progressive neurodegenerative disease with accompanying perceptive disorder. We previously reported that decreasing levels of brain‐derived neurotrophic factor (BDNF) promoted beta‐amyloid (Aβ)‐induced neuronal cell death in neuron‐like differentiated SH‐SY5Y (ndSH‐SY5Y) human neuroblastoma cells in an AD mimic cell model. We investigated the neuroprotective effects of passion fruit seed extract (PFSE) and one of the main stilbene compounds, piceatannol, in an AD cell model using ndSH‐SY5Y cells. Both PFSE and piceatannol were found to protect Aβ‐induced neurite fragmentation in the cell model (protection efficacy; 34% in PFSE and 36% in piceatannol). In addition, both PFSE and piceatannol suppress Aβ‐induced neuronal cell death in the cell model (inhibitory effect; 27% in PFSE and 32% in piceatannol). Our study is the first to report that piceatannol‐rich PFSE can repress Aβ‐induced neuronal cell death by protecting against neurite fragmentation in the AD human cell model. These findings suggest that piceatannol‐rich PFSE can be considered a potentially neuroprotective functional food for both prevention and treatment of AD.

## Graphical abstract

We investigated the neuroprotective effect of piceatannol and passion fruit seed extract (PFSE) rich in this compound in an Alzheimer's disease (AD) mimic cell model. Both piceatannol and PFSE were found to protect beta‐amyloid‐induced neurite fragmentation in the AD cell model. Both piceatannol and PFSE were proven to suppress beta‐amyloid‐induced neuronal cell death in the AD cell model.

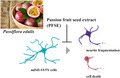

## 1 INTRODUCTION

Alzheimer's disease (AD) is a progressive neurodegenerative disease with accompanying perceptive dysfunction, irreversible memory loss, disorientation, and language impairment (Lane et al., 2018; Liu‐Seifert et al., 2015; Scheff et al., 2006). Beta‐amyloid (Aβ) accumulation is widely considered the main neurotoxic injury that causes AD (Duyckaerts et al., 2009; Fricker et al., 2018; Lane et al., 2018; Mucke & Selkoe, 2012; Scheltens et al., 2016). The common neuropathological features of AD include neurofibrillary tangles comprising hyperphosphorylated tau and senile plaques deposited on Aβ peptides (Duyckaerts et al., 2009; Fricker et al., 2018; Lane et al., 2018; Scheltens et al., 2016). In patients with AD, both brain and serum levels of brain‐derived neurotrophic factor (BDNF)—a neurotrophin widely distributed in adult brains—have been reported to be significantly decreased compared with healthy individuals (Connor et al., 1997; Ng et al., 2019). BDNF plays an important role in the pathophysiology of Aβ‐induced neurotoxicity in AD (Acheson et al., 1995; Arancio & Chao, 2007). We previously demonstrated that the decrease in BDNF triggers self‐aggregated Aβ‐induced neurite fragmentation as well as two types of neuronal cell death, namely caspase‐6‐dependent apoptosis and necroptosis, in an AD mimic cell model using neuron‐like differentiated SH‐SY5Y (ndSH‐SY5Y) human neuroblastoma cells (Tagai et al., 2020). In addition, we have been studying the neuroprotective effects of several natural products and compounds by using an AD mimic cell model.

Passion fruit (*Passiflora edulis* Sims) is a tropical plant belonging to the *Passifloraceae* family, whose fruits are commonly consumed across the world because of its attractive aroma, flavor, and taste (He et al., 2020). Passion fruit seed extract (PFSE) has a potential to become a functional food because of being a rich source of stilbenes, especially piceatannol (3,3′,4′,5‐*trans*‐tetrahydroxy‐stilbene) (Matsui et al., 2010). Piceatannol is a naturally occurring stilbene derivative and a structurally related polyphenol analog of resveratrol (3,4′,5‐*trans*‐trihydroxy‐stilbene). Several previous studies showed that piceatannol possessed neuroprotective effects. Monti et al. (Rivière et al., 2007) indicated that stilbenes, including resveratrol and piceatannol, inhibited Aβ peptide aggregation in vitro. Kim et al. (2007) reported that piceatannol prevented Aβ‐induced reactive oxygen species (ROS) accumulation and apoptosis in PC12 rat pheochromocytoma cells. In addition, Lu et al. (2016) indicated that two stilbenoids, namely piceatannol and pterostilbene, activated the PI3K/Akt/Bad signaling pathway and suppressed both Aβ‐induced ROS accumulation and apoptotic cell death in PC12 cells. Furthermore, a previous study has shown that stilbenoids, including resveratrol and piceatannol, suppress both Aβ‐induced neurotoxicity and cell death by decreasing intracellular ROS via the PI3K/Akt signaling pathway in rat primary cortex neurons (Wen et al., 2018). Although piceatannol has been reported to possess neuroprotective effects, these effects have not been studied before on PFSE. In this study, we have focused on the bioactivities of piceatannol and piceatannol‐rich PFSE as a functional food in both healthcare and preventive care. We previously reported that piceatannol and/or PFSE showed a variety of biological activities such as skin protection (Maruki‐Uchida et al., 2013; Matsui et al., 2010), vasodilatation (Kinoshita et al., 2013), chronic diseases' prevention (Kawakami et al., 2014), and metabolic improvement (Kitada et al., 2017; Uchida‐Maruki et al., 2015; Yamamoto et al., 2017). Interestingly, in our previous study, we indicated that PFSE has suppressed cancer cell proliferation more intensely than piceatannol in both NCI‐H522 human non‐small‐cell lung cancer cells and HCT116 human colorectal cancer cells (Yamamoto et al., 2019).

In this study, we analyzed the neuroprotective effects of piceatannol‐rich PFSE and piceatannol in an AD cell model using human neuroblastoma neuron‐like differentiated cell model (ndSH‐SY5Y cells). In addition, we discussed the potentially neuroprotective functional foods prepared using PFSE in AD prevention and treatment.

## 2 MATERIALS AND METHODS

### 2.1 Reagents

Piceatannol was purchased from Tokyo Chemical Industry. Piceatannol was stored in 100 mM stocks in dimethyl sulfoxide (DMSO, Sigma‐Aldrich; Merck KGaA) at −20°C. All‐*trans*‐retinoic acid (RA) and human‐recombinant animal‐free BDNF were obtained from FUJIFILM Wako Pure Chemical Corporation. Aβ peptide 1–42, *O*‐acyl isopeptide, was purchased from Peptide Institute Inc.

### 2.2 PFSE preparation

Piceatannol‐rich PFSE was prepared as previously described (Yamamoto et al., 2019). Briefly, passion fruit seeds were milled, lyophilized, and extracted with 35% ethanol. After centrifuging the extract, the supernatant was evaporated and lyophilized to obtain a PFSE powder. PFSE, dissolved in DMSO, was stored in 100 mg/ml stocks at −20°C.

### 2.3 Cell culture and cell differentiation

The SH‐SY5Y human neuroblastoma cell line (ATCC^®^ CRL‐2266^TM^) was purchased from the American Type Culture Collection. Cell culture and differentiation were performed as described previously (Tagai et al., 2020). In brief, SH‐SY5Y cells were cultured in Dulbecco's modified eagle medium (D‐MEM)/Ham's F‐12 (FUJIFILM Wako Pure Chemical Corporation, Cat#048‐29785) containing 10% fetal bovine serum (FBS), 100‐units/ml penicillin, and 100‐μg/ml streptomycin in a 37°C incubator in an atmosphere of 5% CO_2_ and 100% relative humidity. In the cell differentiation, SH‐SY5Y cells were inoculated at an initial density of 1.0 × 10^5^ cells/dish in a collagen I‐coated φ 3.5 cm dishes. All‐*trans*‐RA (FUJIFILM Wako) was added 2 days after plating at a final concentration of 10 μM dissolved in high‐glucose D‐MEM (FUJIFILM Wako, Cat#043‐30085), which was supplemented with 15% FBS. After 5 days of culturing in the presence of RA, cells were washed with high‐glucose D‐MEM and incubated for 2 days with 100 ng/ml human recombinant BDNF (FUJIFILM Wako) in high‐glucose D‐MEM without L‐glutamine and phenol red (FUJIFILM Wako, Cat#040‐30095) containing 4 mM sodium pyruvate and 1 mM L‐glutamine. We prepared ndSH‐SY5Y cells using a modified differentiation protocol according to previously described methods (Agholme et al., 2010; Encinas et al., 2000; Krishtal et al., 2017).

### 2.4 Cell viability

Neuron‐like differentiated SH‐SY5Y (i.e., ndSH‐SY5Y) cells in several developmental stages were stained with 7‐aminoactinomycin D (7‐AAD; Thermo Fisher Life Technologies) and Hoechst33342 (Thermo Fisher Life Technologies). Cell images were analyzed using a LAS AF microscope coupled with a Leica DMI6000B‐AFC system at ×200 magnification, and calculations were performed based on the proportion of 7‐AAD‐positive cells.

### 2.5 Morphological observation

Cell morphology was observed under a Leica DMi1 microscope using a LAS v4.12 platform at ×200 magnification. Measurements of both neurite length and neurite fragmentation were analyzed using the image processing software ImageJ using the NeuronJ plugin and Particle Analysis. Neurite fragments were measured as follows: image binarization followed by automatic calculation by particle analysis using appropriate parameters (size: 0.00005–0.0005; circularity: 0.40–1.00).

### 2.6 Statistical analysis

Statistical analysis was performed using GraphPad Prism 9 software. Data were presented as means ±standard deviation (*SD*). The significance of the differences among groups was determined using the Student's *t*‐test and one‐way ANOVA. *p* < .05 was considered as significant.

## 3 RESULTS

### 3.1 PFSE and piceatannol prevent Aβ‐induced neurite fragmentation

**FIGURE 1:**
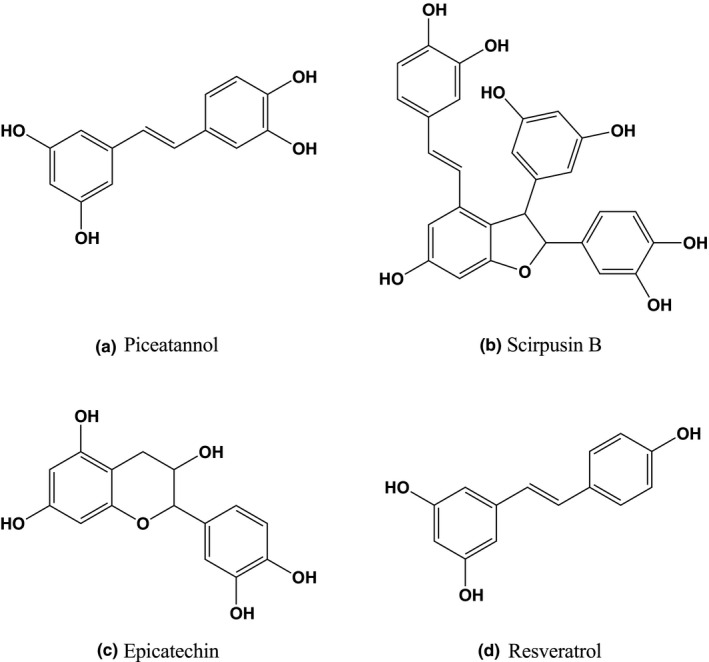
Chemical structures of main polyphenol content in passion fruit seed extract. (a) Piceatannol. (b) Scirpusin B. (c) Epicatechin. (d) Resveratrol

**FIGURE 2:**
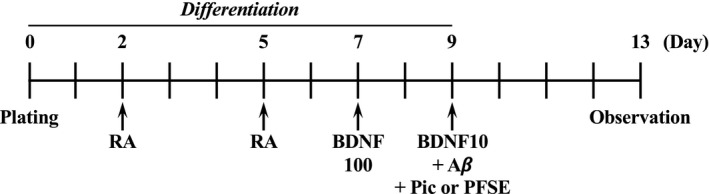
Schedule of neuron‐like differentiation of SH‐SY5Y cells and overview of the experimental Alzheimer's cell model. RA, 10 μM RA; 100B, 100 ng/ml BDNF; 10B, 10 ng/ml BDNF; Aβ, 3 µM Aβ; Pic, 1 µM piceatannol; PFSE, 2.4 µg/ml PFSE (piceatannol 1 μM equivalent)

We previously reported an AD mimic cell model using ndSH‐SY5Y cells (Table 1) (Tagai et al., 2020). We also showed that both Aβ‐induced neurite fragmentation and cell death modes were modified under reducing concentrations of BDNF (Tagai et al., 2020). In this study, to examine the neuroprotective effects of piceatannol‐rich PFSE and piceatannol, we analyzed their effects on Aβ‐induced neurite fragmentation and cell death in an AD cell model using ndSH‐SY5Y cells. PFSE was found to contain piceatannol, as the most abundant polyphenol, at a concentration of 104.5 μg/mg (Table 2) (Yamamoto et al., 2019). Figure 1 indicates the chemical structures of the main polyphenol content in PFSE. In addition, Figure 2 shows the scheme of experimental procedure in the ndSH‐SY‐5Y cell model. This evaluation method differentiates cells and is not suitable for the CCK‐8 assay on a 96‐well plate. Cells were treated for 5 days (days 2–7) with 10‐μM RA before incubation with 100 ng/ml BDNF for 2 days (days 7–9). On the basis of the morphological observations, ndSH‐SY5Y cells ceased to proliferate on day 5, and their morphology became neuron like, including the development of long neurites that were visible on days 5–9 (Tagai et al., 2020). It should be noted that the neurite length of ndSH‐SY5Y cells was approximately 87 μm on day 9 (Tagai et al., 2020). On day 9, ndSH‐SY5Y cells expressed growth‐associated protein 43 (GAP43), a neuron‐specific marker protein (Table 1) (Tagai et al., 2020).

**TABLE 1:** The biological and morphological features of AD mimic cell model

Cell line	SH‐SY5Y human neuroblastoma cells
Differentiation	Yes (RA and BDNF)
Neuronal marker	GAP43 positive
Tested cell type	Neuron‐like differentiated SH‐SY5Y cells
Stimulus	BDNF decreasing and Aβ peptide 1–42
AD mimic cell model	
Morphological feature	Neurite fragmentation
Cell death mode	Caspase−6‐dependent apoptosis and necroptosis
Response to cell death inhibitors	
Pan‐caspase inhibitor (Z‐VAD‐FMK, 100 μM)	Inhibited
Caspase−6 inhibitor (Z‐VEID‐FMK, 100 μM)	Inhibited
Necroptosis inhibitor (Necrostatin−1, 20 μM)	Inhibited

Abbreviations: BDNF, brain‐derived neurotrophic factor; GAP43, growth‐associated protein 43; RA, retinoic acid.

**TABLE 2:** Polyphenol content of passion fruit seed extract (PFSE)

Name	Volume (mg/g in PFSE)
Piceatannol	104.5
Scirpusin B	45.5
Epicatechin	0.99
Resveratrol	0.082
Isorhapontigenin	0.013
Astringin	0.0068
*p*‐Coumaric acid	0.0023
Caffeic acid	0.00082
Piceid	0.00070
Pinostilbene	≥0.00014

Note

Polyphenols present in PFSE were analyzed as described previously (Yamamoto et al., 2019). Briefly, PFSE was analyzed via multiple‐reaction monitoring combined with ultrafast liquid chromatograph and electrospray ionization tandem mass spectrometry system.

**FIGURE 3:**
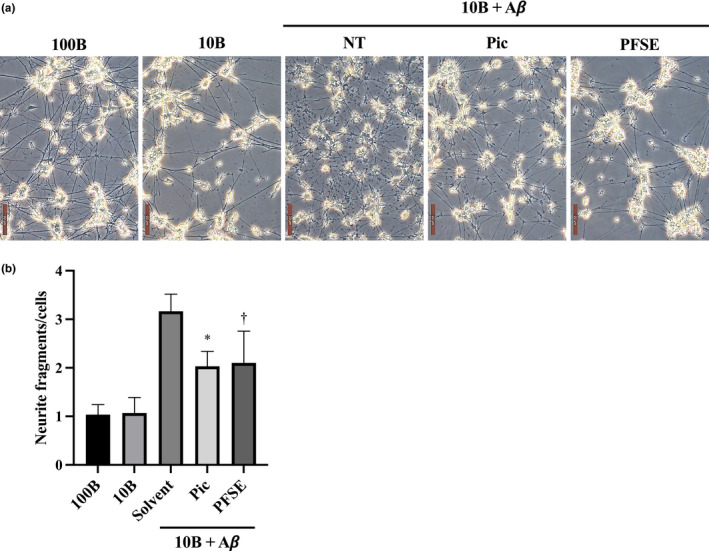
Passion fruit seed extract (PFSE) prevents Aβ‐induced neurite fragmentation in ndSH‐SY5Y cells. (a) Morphology of the ndSH‐SY5Y cells under the indicated conditions for 4 days (on day 13). 100B, cells were treated with 100 ng/ml BDNF; 10B, cells were treated with 10 ng/ml BDNF; 10B + Aβ, cells were cotreated with 10 ng/ml BDNF and 3 µM Aβ; vehicle, solvent alone; Pic, cells were supplemented with 1 μM piceatannol; PFSE, cells were supplemented with 2.4 µg/ml PFSE. Scale bar =100 μm. (b) Neurite fragmentation of ndSH‐SY5Y cells under the indicated conditions for 96 h (from day 9 to day 13). Results represent averages of three independent experiments, with error bars showing ± *SD*. *p* = .0003 (ANOVA), * *p* = .0135 (vs. solvent), †*p* = .0679 (vs. solvent). Neurite length of ndSH‐SY5Y cells on day 13 under the indicated culture conditions for 96 h from day 9. 100B, cells were treated with 100 ng/ml BDNF; 10B, cells were treated with 10 ng/ml BDNF; 10B + Aβ, cells were cotreated with 10 ng/ml BDNF and 3 µM Aβ; NT, indicated vehicle, solvent alone; Pic, cells were supplemented with 1 μM piceatannol; PFSE, cells were supplemented with 2.4 µg/ml PFSE

At first, we examined the protective effects of PFSE and piceatannol on neurite fragmentation in an AD cell model. Neurite fragmentation was only scarcely observed when BDNF levels were decreased (Figure 3). Thus, treatments of ndSH‐SY5Y cells with either 10 or 100 ng/ml BDNF for 96 h on day 9 did not change cells’ morphology at day 13 (Figure 3a). As shown in Figure 3, neurite fragmentation was dramatically increased by the treatment with Aβ for 96 h under decreased BDNF levels as compared with the treatments containing normal BDNF levels. Under low levels of BDNF, neurite fragmentation in the Aβ‐treated ndSH‐SY5Y cells was suppressed by the treatment with either PFSE or piceatannol (Figure 3). Previously, numerous studies on neuroprotective activities have evaluated the bioactivity of piceatannol in the 1–30 μM concentration range (Arai et al., 2016; Fu et al., 2016; Kim et al., 2007). In addition, we have been investigating the neuroprotective effects of several natural compounds and synthetic compounds at approximately 1 μM concentration in an AD mimic cell model. Therefore, this cell model was used to test the biological activity of PFSE and piceatannol at 2.4 μg/ml (piceatannol 1 μM equivalent) and 1 μM, respectively. We found that PFSE and piceatannol suppressed Aβ‐induced neurite fragmentation in ndSH‐SY5Y cells at similar levels. This finding suggests that both piceatannol‐rich PFSE and piceatannol can prevent Aβ‐induced neurite fragmentation in an AD cell model.

### 3.2 PFSE and piceatannol suppress Aβ‐induced neuronal cell death

**FIGURE 4:**
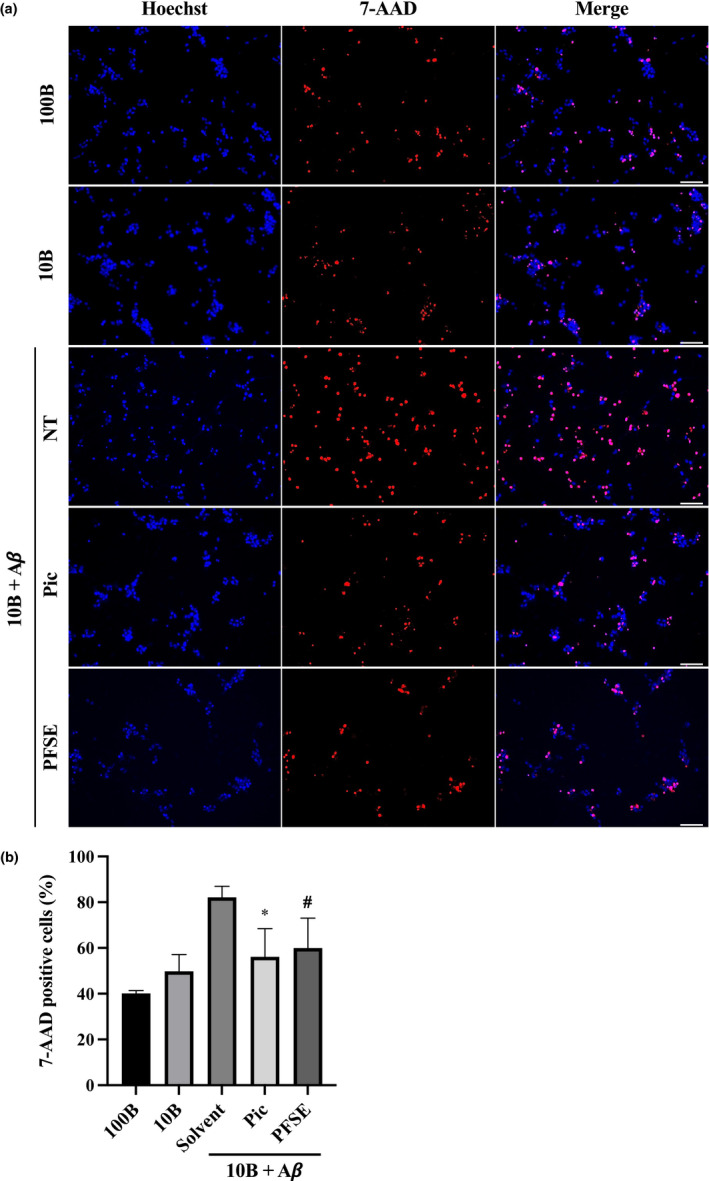
Passion fruit seed extract (PFSE) suppresses Aβ‐induced cell death in ndSH‐SY5Y cells. (a) Images of Hoechst‐ and 7‐AAD‐stained ndSH‐SY5Y cells under the indicated conditions for 4 days (on day 13). 100B, cells were treated with 100 ng/ml BDNF; 10B, cells were treated with 10 ng/ml BDNF; 10B + Aβ, cells were cotreated with 10 ng/ml BDNF and 3 µM Aβ; vehicle, solvent alone; Pic, the cells were supplemented with 1 μM piceatannol; PFSE, cells were supplemented with 2.4 µg/ml PFSE. Scale bar =50 μm. Two additional independent experiments gave similar results. (b) The 7‐AAD‐positive dead cells’ proportion (%) of ndSH‐SY5Y cells under the indicated conditions on day 13. These data were obtained by counting more than 250 cells (10 pictures) in each independent microscopic examination. Results represent averages of three independent experiments, with error bars showing ±*SD* (# indicates two independent experiments). *p* = .002 (ANOVA), * *p* = .0275 (vs. solvent), # *p* = .0654 (vs. solvent). Dead cells of ndSH‐SY5Y on day 13 under the indicated culture conditions for 96 h from day 9. 100B, cells were treated with 100 ng/ml BDNF; 10B, cells were treated with 10 ng/ml BDNF; 10B + Aβ, cells were cotreated with 10 ng/ml BDNF and 3‐µM Aβ; NT, indicated vehicle, solvent alone; Pic, cells were supplemented with 1 μM piceatannol; PFSE, cells were supplemented with 2.4 µg/ml PFSE

In a previous study, we demonstrated that neuronal cell death, induced by both aggregated Aβ and the low levels of BDNF, is characterized as caspase‐6‐dependent apoptosis and necroptosis in an AD cell model (Table 1) (Tagai et al., 2020). Here, we investigated the inhibitory effect of PFSE and piceatannol on neuronal cell death in an AD cell model using ndSH‐SY5Y cells. Neuronal cell death, indicated by the 7‐AAD‐positive cells, slightly increased by decreasing BDNF levels as compared with the normal BDNF levels (Figure 4). As shown in Figure 4, the proportion of neuronal cell death increased by 82% after the treatment with Aβ for 96 h under the decreased BDNF conditions. Meanwhile, the proportion of 7‐AAD‐positive dead cells in the treated ndSH‐SY5Y cells was significantly decreased by approximately 60% or 56% after the treatment with PFSE or piceatannol, respectively (Figure 4). We also showed that PFSE and piceatannol depress Aβ‐induced ndSH‐SY5Y cell death at similar levels. These results indicated that both piceatannol‐rich PFSE and piceatannol were able to suppress Aβ‐induced neuronal cell death in an AD cell model.

## 4 DISCUSSION

Numerous studies have been performed on functional foods for Alzheimer's disease, namely foods or nutrients that provide medical or health benefits, including prevention and treatment of the disease (Atlante et al., 2020). Several functional foods and nutraceutical compounds act at a variety of biochemical and metabolic levels, and much evidence shows their neuroprotective effects in fundamental studies, that is, in vitro and in vivo experiments (Atlante et al., 2020).

In this study, we focused on the biological effects of piceatannol‐rich PFSE and piceatannol in order to be evaluated as a functional food in healthcare, preventive care, and diverse chronic disease treatment. It was previously suggested that PFSE powder has the potential to be used as a functional food, being a rich source of stilbenes, especially piceatannol (Matsui et al., 2010). In the present study, we showed that both piceatannol and piceatannol‐rich PFSE were able to prevent Aβ‐induced neurite fragmentation and neuronal cell death in an AD cell model using neuron‐like differentiated human SH‐SY5Y cells. We also found that the protecting effects of PFSE and piceatannol on the Aβ‐induced neurotoxicity were similar. It is worth noting that our study provides the first evidence that piceatannol‐rich PFSE repressed Aβ‐induced neuronal cell death via the protection of neurite fragmentation in an AD cell model. Notably, PFSE is very attractive as a functional food because it contains various polyphenols such as scirpusin B, epicatechin, and resveratrol in addition to piceatannol. These findings suggest that PFSE can be used as a potential neuroprotective functional food in AD prevention and treatment.

Several previous studies demonstrated that piceatannol exhibited neuroprotective effects on Aβ‐induced neurotoxicity in both rat pheochromocytoma PC12 cells and rat primary cortex neurons (Fu et al., 2016; Kim et al., 2007; Wen et al., 2018). In the amyloid cascade hypothesis of AD onset, deposition of neurotoxic Aβ in the brain is regarded as an important reason of AD pathogenesis (Lu et al., 2013). Aβ peptides are processed by the proteolysis of the amyloid precursor protein by β‐ and γ‐secretase (Benilova et al., 2012). Many previous studies have confirmed that Aβ neurotoxicity is associated with oxidative stress (Butterfield et al., 2007; Cai et al., 2011; Cheignon et al., 2018). As reported previously, piceatannol inhibited both Aβ‐induced ROS accumulation and caspase‐3‐dependent apoptosis in PC12 cells (Kim et al., 2007). In addition, Li et al. (Fu et al., 2016) demonstrated that piceatannol repressed both Aβ‐induced apoptosis, mediating the PI3K/Akt/Bad signaling and downstream mitochondria‐caspase‐3‐dependent cell death pathway in PC12 cells. Furthermore, piceatannol suppressed Aβ‐induced neurotoxicity by decreasing intracellular ROS accumulation via the PI3K/Akt signaling pathway in rat primary cortex neurons (Wen et al., 2018) and prevented Aβ‐induced intracellular ROS accumulation in both rat PC12 cells and rat primary cortex neurons (Fu et al., 2016; Wen et al., 2018). Interestingly, our collaborators previously revealed that piceatannol promoted neural stem cell differentiation into astrocytes (Arai et al., 2016). Notably, oral administration of piceatannol increased the proportion of astrocytes in the brains of adult mice (Arai et al., 2016). These findings suggest that piceatannol can successfully pass through the blood–brain barrier. Importantly, we further investigated the neuronal cell death mode(s) and the detail action mechanisms of PFSE in an AD mimic cell model. In this regard, we are currently investigating cell death and amyloidosis marker proteins in an AD cell model via comprehensive proteomic analysis. We also realize that additional primary human neuronal cell‐based and in vivo animal studies are warranted to verify the efficacies of PFSE and piceatannol.

In conclusion, piceatannol‐rich PFSE can be considered a promising neuroprotective functional food in the maintenance of cerebral function as well as in the prevention and treatment of AD.

## CONFLICT OF INTEREST

Akira Sato and Sei‐ichi Tanuma received a research grant from Morinaga & Co., Ltd. Shinpei Kawakami, Takayuki Yamamoto, Hiroko Maruki‐Uchida, Sadao Mori, and Minoru Morita are employees of Morinaga & Co., Ltd.

## DATA AVAILABILITY STATEMENT

The data that support the findings of this study are available from the corresponding author upon reasonable request.
